# The After-Care Association for the Insane

**Published:** 1899-05-27

**Authors:** 


### The Institutional Workshop.

### THE AFTER-CARE ASSOCIATION FOR THE INSANE.

Few forms of disease are more pitiable than insanity, and few have so little thought bestowed on them by the charitable. While the patient is an inmate of an asylum his liberty is taken from him, and when he is discharged recovered from it his troubles are by no means over, for too often he finds that his old companions look askance at him, and that his former employer knows him not. This want of sympathy, and this absence of work, the latter bringing with it not infrequently want of proper food, coming at a time when the balance of mind is still unstable, must be peculiarly hard to bear. It is the praiseworthy object of the After-care Association to mitigate all these evils; but it is sadly cramped in its endeavours by insufficient funds; but we would fain believe that this in great part is owing to the fact that the association is not widely known. Last year its funds amounted only to £652; and there were 186 cases brought before the council as suitable for relief. The officers of the association thoroughly investigate every case in which aid is asked for; and it may be imagined that much difficulty is involved in these investigations, as a dozen visits may have to be made in one case. Some ex-patients are boarded out in country cottages; some have given to them grants of money or clothing; and, above all, some have occupation found for them. Mr. Thornhill Roxby is the secretary, and the offices are at Church House, Dean's Yard, Westminster. An annual subscription of 5s. constitutes an ordinary member, and a single subscription of £2 10s. a life member. Donations of any sum will be received.

